# Neural Cell Adhesion Molecule Regulates Osteoblastic Differentiation Through Wnt/β-Catenin and PI3K-Akt Signaling Pathways in MC3T3-E1 Cells

**DOI:** 10.3389/fendo.2021.657953

**Published:** 2021-05-12

**Authors:** Bin-Feng Cheng, Xiao Feng, Yao-Xin Gao, Shao-Qin Jian, Shi-Rao Liu, Mian Wang, Yun-Fei Xie, Lei Wang, Zhi-Wei Feng, Hai-Jie Yang

**Affiliations:** ^1^ School of Life Sciences and Technology, Xinxiang Medical University, Xinxiang, China; ^2^ Institute of Precision Medicine, Xinxiang Medical University, Xinxiang, China; ^3^ Henan Children’s Hospital, Zhengzhou, China

**Keywords:** NCAM, osteoblast differentiation, Wnt/β-catenin signaling, PI3K-Akt signaling, MC3T3-E1 cells

## Abstract

Neural cell adhesion molecule (NCAM) is involved in cell multi-directional differentiation, but its role in osteoblast differentiation is still poorly understood. In the present study, we investigated whether and how NCAM regulates osteoblastic differentiation. We found that NCAM silencing inhibited osteoblast differentiation in pre-osteoblastic MC3T3-E1 cells. The function of NCAM was further confirmed in NCAM-deficient mesenchymal stem cells (MSCs), which also had a phenotype with reduced osteoblastic potential. Moreover, NCAM silencing induced decrease of Wnt/β-catenin and Akt activation. The Wnt inhibitor blocked osteoblast differentiation, and the Wnt activator recovered osteoblast differentiation in NCAM-silenced MC3T3-E1 cells. We lastly demonstrated that osteoblast differentiation of MC3T3-E1 cells was inhibited by the PI3K-Akt inhibitor. In conclusion, these results demonstrate that NCAM silencing inhibited osteoblastic differentiation through inactivation of Wnt/β-catenin and PI3K-Akt signaling pathways.

## Introduction

Osteoporosis (OP) is one of the most common bone disorders in human beings, its incidence increases with age, especially in postmenopausal women because of the decrease in estrogen (1, 2). With the growth of the aging population in the world, the prevalence of OP is increasing dramatically, bringing a huge economic burden to the society and families (3, 4).

OP is characterized by a reduction in bone mineral density, which is mainly due to the imbalance of bone tissue formation and absorption (5, 6). Bone homeostasis depends on the balance between osteoblasts and osteoclasts, OP occurs as a result of the decrease of osteoblast-induced bone formation and the increase of osteoclast-induced bone absorption. Bone weakness and fracture are common in patients with OP because of their low bone mass and quality. Abnormal proliferation and differentiation of osteoblasts were shown involved in reduction of bone mass (7, 8). Study on the molecular mechanisms of osteoblastic differentiation is helpful to understand the pathogenesis of OP and develop new strategies for OP treatment.

Neural cell adhesion molecule (NCAM, CD56), a member of the immunoglobulin (IgG) superfamily, is widely expressed in the nervous system and plays multi-functional roles in neural regeneration, cell to cell adhesion, signal transduction and modulation of learning and memory (9–11). NCAM has been found to be expressed in other cell types such as epithelial cells, natural killer cells, skeletal muscle and mesenchymal stem cells (MSCs) (12, 13). In our previous studies, we demonstrated that NCAM enhances insulin sensitivity and promotes adipocyte differentiation, and we further found that NCAM inhibits hypertrophic chondrocyte differentiation and reduces chondrocyte hypertrophy in experimental osteoarthritis model (14, 15). Our results indicate that NCAM plays a key role in cell multi-directional differentiation, however, the function of NCAM in osteoblastic differentiation remains largely unknown.

In the present study, we evaluated the effects of NCAM on osteoblastic differentiation in mouse preosteoblast-like cells (MC3T3-E1) and in mouse MSCs. We also investigated the underlying mechanism involved in NCAM-regulated osteoblastic differentiation.

## Materials and Methods

### Materials

LY294002, β-glycerophosphate, ascorbic acid-2-phosphate, and dexamethasone were obtained Sigma-Aldrich (St. Louis, MO, USA). XAV939 was obtained from EMD Millipore (Darmstadt, Germany). Lithium Chloride (LiCl) was purchased from Nacalai Tesque, Inc. (Kyoto, Japan). Fetal bovine serum (FBS) was purchased from Gibco (Grand Island, NY, USA). The mammalian expression vector pcDNA4/Myc was obtained from Invitrogen (Carlsbad, CA, USA). siRNA expression vectors pSilencer 4.1-CMV neo was obtained from Ambion (Austin, TX, USA). Lipofectamine 2000, G418 and puromycin were purchased from Invitrogen (Carlsbad, CA, USA). Antibodies against RunX2, Osterix, β-catenin, phospho-Akt, and β-actin were purchased from Cell Signaling Technology, Inc. (Beverly, MD, USA). The secondary antibodies were purchased from Santa Cruz Biotechnology (Santa Cruz, CA, USA). TRIzol regent was purchased from Invitrogen (Carlsbad, CA, USA). QuantiTect Reverse Transcription kit was purchased from Qiagen (Valencia, CA, USA). SYBR Green Master Mix was purchased from Bio-Rad Laboratories (Richmond, CA, USA). Other chemicals were purchased from Sigma-Aldrich.

### NCAM-Deficient Mice and MSCs

The *Ncam^–/–^* (knockout; KO) mice were generated on a C57/BL6 background as previously described (16). Wild-type (WT) and KO MSCs were obtained from 8-week-old male mice as previously described (14). Briefly, cells were harvested from mouse bone marrow and cultured in low glucose Dulbecco’s modified Eagle’s medium (DMEM-LG, with 1 g/L glucose; Hyclone, Logan, UT, USA) containing 10% fetal bovine serum (FBS), 100 IU/mL penicillin and 100 g/mL streptomycin. Non-adherent hematopoietic cells were discarded after incubation for 7 days, and the adhered MSCs were purified by repeated passaging. MSCs with fibroblast-like morphology from passage 6 to 9 was used in this study.

### Cell Culture and Osteogenic Differentiation

The mouse osteoblastic cell line MC3T3-E1 was purchased from American Type Culture Collection (ATCC; Rockville, MD, USA). Cells were cultured in α-modified Eagle’s medium (α-MEM; Hyclone, Logan, UT, USA) supplemented with 10% FBS, penicillin (100 U/mL), and streptomycin (100 g/mL).

WT and KO MSCs were cultured in DMEM-LG containing 10% FBS, 100 IU/mL penicillin and 100 g/mL streptomycin as previously described (14).

Cells were seeded in 12-well plates at a concentration of 1×10^5^ cells/mL. The MC3T3-E1 cells and MSCs were respectively incubated in a-MEM and DMEM with 10 mM β-glycerophosphate, 25 µM ascorbic acid-2-phosphate and 10 nM dexamethasone.

### Plasmids and Transfection

siRNA vectors silencing NCAM and plasmids expressing full-length mouse NCAM were designed and constructed as previously described (14). Transfection was conducted using Lipofectamine 2000 following manufacturer’s instructions. To obtain stable mixed cell lines, cells were selected with puromycin at 1 μg/mL and G418 at 800 µg/mL for 14 days. Gene silencing or overexpression of NCAM was validated by Western blotting with anti-NCAM antibody.

### Real-Time PCR

Total RNA was extracted with TRIzol (Invitrogen) according to the manufacturer’s instructions, and cDNA was synthesized using a High-Capacity cDNA Reverse Transcription Kit (Applied Biosystems, Foster City, CA). The mRNA levels were measured using an ABI Prism 7500 Sequence detection system (Applied Biosystems) and SYBR Green qPCR Master Mix (KAPA Biosystems). The expression levels of *RunX2* and *Osterix* were normalized using the internal reference gene *GAPDH*. The PCR primers were shown in **Table 1**.

**Table 1:** Primers for real-time PCR.

Gene	Forward primer (5’-3’)	Reverse primer (5’-3’)
RunX2	CGAAATGCCTCCGCTGTTAT	TGAGGAATGCGCCCTAAATC
Osterix	TGACTACCCACCCTTCCCTC	GCCTTGTACCACGAGCCATA
GAPDH	CTTCAACAGCAACTCCCACT	GTCCAGGGTTTCTTACTCCT

### Western Blot

Cells were lysed with radio immuno-precipitation (RIPA) buffer and lysed on ice supplemented with proteinase and phosphatase inhibitors cocktail (Sigma). Protein concentrations were determined using BCA Protein Assay Kit (Pierce). Equal amounts of protein were separated by sodium dodecyl sulfate-polyacrylamide gel electrophoresis and transferred to polyvinylidene difluoride (PVDF) membrane. After blocking with 3% bovine serum albumin (BSA; Sigma) for 1 h, the membranes were incubated with primary antibodies against RunX2, Osterix, β-catenin, p-Akt, or β-actin overnight at 4°C. The membranes subsequently were washed three times with TBST and incubated with the secondary antibodies for 40 min at room temperature. The band density was scanned by ImageQuant LAS 4000 system (GE Healthcare) and analysed by ImageJ software.

### Alizarin Red Staining and Quantification

The alizarin red staining was performed at 0, 7, or 14 days. Cells were fixed in 4% paraformaldehyde (PFA) for 30 min followed by staining with 40 mM Alizarin Red S (ARS; pH 4.2) for 30 min and then washed three times with deionized water. For the quantitative analysis of ARS, the stained wells were incubated with 10% cetyl pyridinium chloride monohydrate for 30 min at room temperature and then measured at 562 nm using a Molecular Devices microplate reader (15).

### Statistical Analysis

All data are expressed as the mean ± SEM. Statistical analysis was performed using one-way ANOVA followed by Tukey’s test. Differences were regarded as statistically significant at P < 0.05. Each experiment was performed at least in triplicate.

## Results

### NCAM Silencing Inhibits Osteoblast Differentiation of MC3T3-E1 Cells

**Figure 1:**
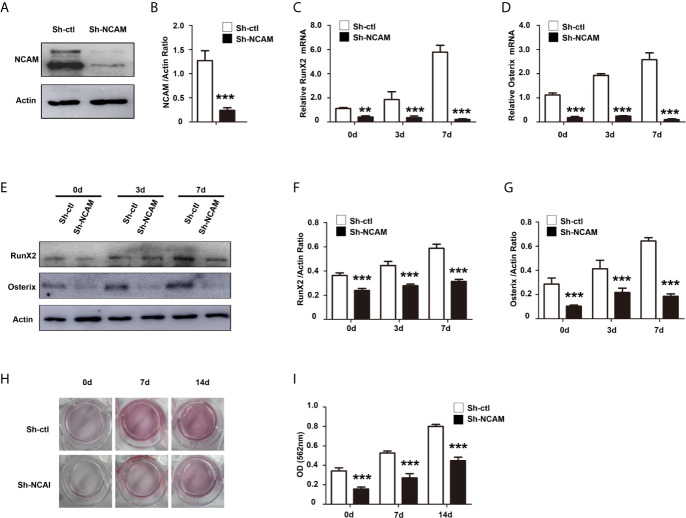
NCAM silencing inhibits osteoblast differentiation of preosteoblast-like MC3T3-E1 cells. **(A)** Cells were transfected with pSilencer-4.1-based plasmid containing a scrambled sequence (control; Sh-ctl) or a 19-bp oligonucleotide insert targeting mouse NCAM (Sh-NCAM), and analysed by immunoblotting with anti-NCAM antibody. **(B)** Level of NCAM was quantified by densitometry and normalized to β-actin (n=3; mean ± SEM; ***p < 0.001, compared with control siRNA group). The expression of RunX2 **(C)** and Osterix **(D)** was analysed by real-time PCR (The results are expressed as the mean ± SEM of three independent experiments. **p < 0.01, ***p < 0.001, compared with control siRNA-transfected cells). **(E)** The expression of RunX2 and Osterix was analysed by immunoblotting, β-actin was detected as a loading control. Levels of **(F)** RunX2 and **(G)** Osterixwere quantified by densitometry and normalized to β-actin (Data are representative of three independent experiments and values are means ± SEM. ***p < 0.001, compared with control siRNA-transfected cells). **(H)** Cells transfected with control or NCAM siRNA were induced with osteoblastic media and stained with Alizarin red. **(I)** The Alizarin red staining was extracted and quantified, the wavelength was measured at 562 nm (Data are representative of three independent experiments and values are means ± SEM. ***p < 0.001, compared with control siRNA-transfected cells).

To explore the function of NCAM in osteoblast differentiation, stable NCAM down-regulated MC3T3-E1 cells were developed using plasmid-based small interfering RNA (siRNA). The expression level of NCAM was determined using western blot. The result showed that NCAM expression in the Sh-NCAM cells was much lower compared with that of Sh-ctl cells and the interference rate reached 70% (**Figures 1A, B**). The gene expressions of osteogenic markers *RunX2* and *Osterix* were examined by quantitative real-time PCR, down-regulation of NCAM inhibited expressions of *RunX2* and *Osterix* during osteogenic differentiation (**Figures 1C, D**). Western blotting analysis showed that the protein levels of RunX2 and Osterix were also decreased in Sh-NCAM MC3T3-E1 cells (**Figures 1E–G**). In addition, calcium deposition was visualized by Alizarin Red staining and quantitative analysis, the calcium content was significantly decreased in Sh-NCAM cells as compared to Sh-ctl MC3T3-E1 cells (**Figures 1H, I**). These results demonstrated that gene silencing of NCAM inhibits osteoblast differentiation of MC3T3-E1 cells.

### NCAM Deficiency Impairs Osteoblast Differentiation of Mouse MSCs

**Figure 2:**
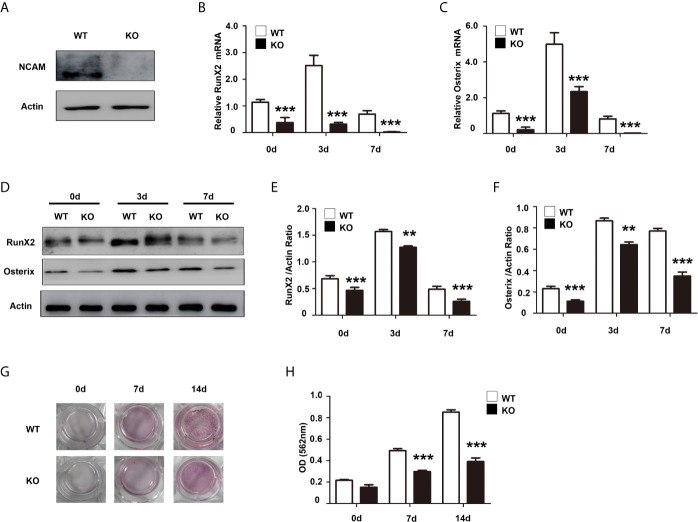
NCAM deficiency inhibits osteoblast differentiation of mouse MSCs. **(A)** Wild-type (WT) and *Ncam^–/–^* (KO) MSCs were examined with anti-NCAM antibody. β-actin was detected as a loading control. The expression of RunX2 **(B)** and Osterix **(C)** was analysed by real-time PCR (The data are expressed as the mean ± SEM of three independent experiments. ***p < 0.001, compared with WT MSCs). **(D)** The expression of RunX2 and Osterix was analysed by immunoblotting, β-actin was detected as a loading control. Levels of RunX2 **(E)** and Osterix **(F)** were quantified by densitometry and normalized to β-actin (Data are representative of three independent experiments and values are means ± SEM. **p < 0.01, ***p < 0.001, compared with WT MSCs). **(G)** Calcium deposition of differentiated WT and KO MSCs was assessed by Alizarin red staining. **(H)** The Alizarin red staining was extracted and quantified, the wavelength was measured at 562 nm (Data are representative of three independent experiments and values are means ± SEM. ***p < 0.001, compared with WT MSCs).

To further confirm the role of NCAM in osteoblast differentiation, we isolated bone marrow derived MSCs from wild-type (WT) and *Ncam^–/–^* (KO) mice. The Western blot results confirmed the expression of NCAM in wild-type MSCs but not in *Ncam^–/–^* cells (**Figure 2A**). To investigate whether NCAM deficiency affects osteoblast differentiation of MSCs, wild-type and *Ncam^–/–^* MSCs were treated with osteogenic differentiation medium. The mRNA expressions of *RunX2* and *Osterix* were down-regulated in *Ncam^–/–^* MSCs as compared to WT cells (**Figures 2B, C**). The protein levels of RunX2 and Osterix were detected by Western blotting. As shown in **Figures 2D–F**, the induction of RunX2 and Osterix in *Ncam^–/–^* MSCs was also lower than that in wild-type cells. Alizarin red staining and quantitative analysis of the calcium content in *Ncam^–/–^* MSCs showed decreased calcium deposition as compared to wild-type cells (**Figures 2G, H**). The results further confirmed the NCAM level involved in osteoblast differentiation of MSCs, suggesting that NCAM might play a key role in osteogenesis of various cell lines.

### Wnt/β-catenin Signaling Contributes to NCAM-Mediated Osteoblast Differentiation

**Figure 3:**
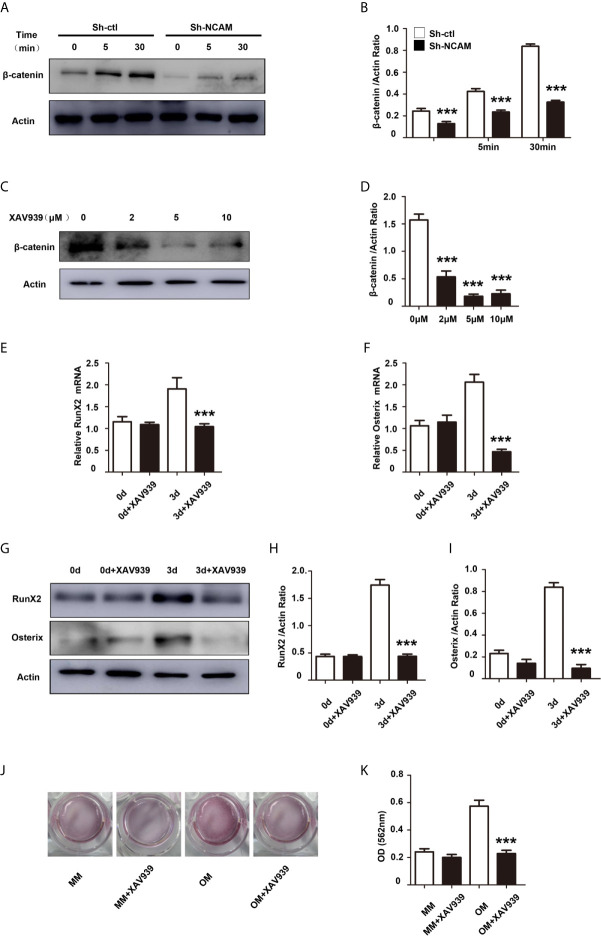
Inhibition of Wnt/β-catenin signaling inhibits osteoblast differentiation. **(A)** Sh-ctl and Sh-NCAM MC3T3-E1 cells were treated with differentiation media for 0, 5, and 30 min, the expression of β-catenin was analysed by immunoblotting. β-actin was detected as a loading control. **(B)** Level of β-catenin was quantified by densitometry and normalized to β-actin (n=3; mean ± SEM; ***p < 0.001, compared with control siRNA group). MC3T3-E1 cells were pretreated with the Wnt inhibitor XAV939. **(C)** The expression of β-catenin was analysed and **(D)** quantified by densitometry and normalized to β-actin (n=3; mean ± SEM; ***p < 0.001, compared with cells without XAV939). The expressions of **(E)** RunX2 and **(F)** Osterix were examined by real-time PCR (The results are expressed as the mean ± SEM of three independent experiments. ***p < 0.001, compared with cells without XAV939). **(G)** The expressions of RunX2 and Osterix were analysed by immunoblotting, β-actin was detected as a loading control. Levels of **(H)** RunX2 and **(I)** Osterix were quantified by densitometry and normalized to β-actin (Data are representative of three independent experiments and values are means ± SEM. ***p < 0.001, compared with cells without XAV939). **(J)** Calcium deposition was assessed by Alizarin red staining. **(K)** The Alizarin red staining was extracted and quantified, the wavelength was measured at 562 nm (Data are representative of three independent experiments and values are means ± SEM. ***p < 0.001, compared with cells without XAV939).

It has been shown that the Wnt/β-catenin signaling pathway plays an important role in osteogenic lineage (17). To investigate the mechanism of osteoblast differentiation mediated by NCAM, the expression of β-catenin was examined in Sh-ctl and Sh-NCAM MC3T3-E1 cells. The Western blotting results showed that β-catenin level was significantly decreased in Sh-NCAM cells (**Figures 3A, B**). To determine the role of β-catenin, we applied the Wnt signaling inhibitor XAV939 during osteogenic differentiation in MC3T3-E1 cells. The β-catenin expression was almost completely inhibited by XAV939 (≥ 5 μM) (**Figures 3C, D**). As a result of β-catenin suppression, the mRNA expressions of *RunX2* and *Osterix* were down-regulated (**Figures 3E, F**). Accordingly, XAV939 at 5 μM totally inhibited the protein levels of RunX2 and Osterix in MC3T3-E1 cells (**Figures 3G–I**). In addition, calcium deposition was also significantly blocked by XAV939 (**Figures 3J, K**). These results indicate that Wnt/β-catenin signaling is involved in NCAM-mediated osteoblast differentiation.

**Figure 4:**
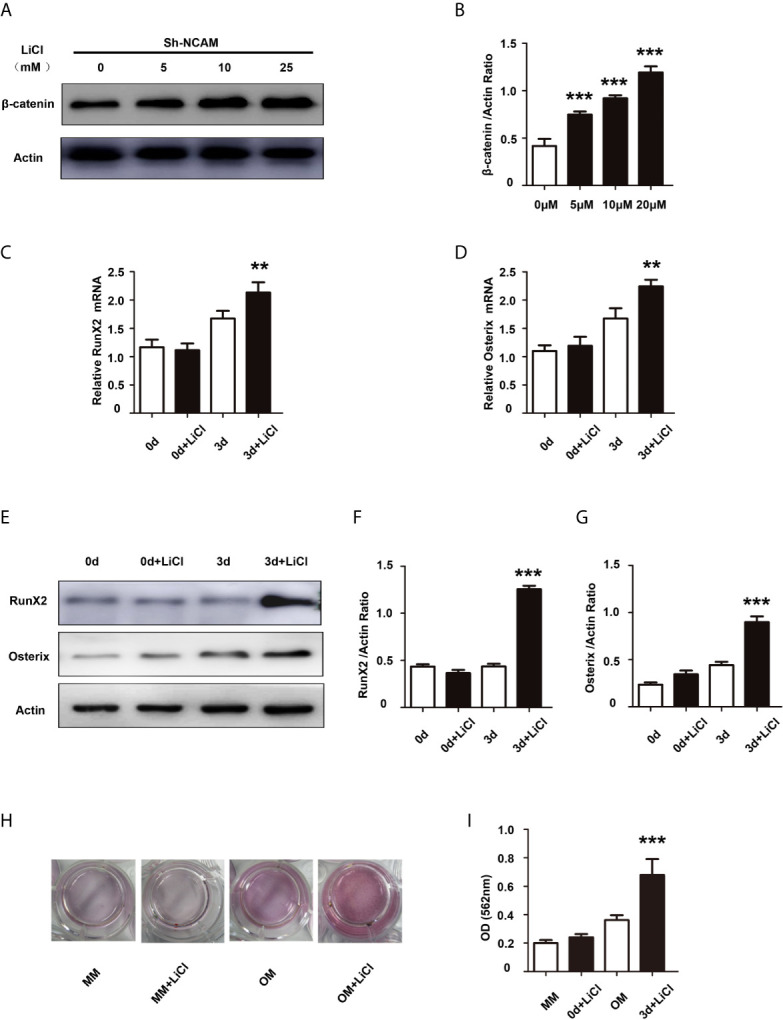
Activation of Wnt/β-catenin signaling recovers osteoblast differentiation inhibited by NCAM silencing. Sh-NCAM MC3T3-E1 cells were pretreated with the Wnt agonist LiCl. **(A)** the expression of β-catenin was analysed by immunoblotting at 0, 5, 10 and 25 min, β-actin was detected as a loading control. **(B)** Level of β-catenin was quantified by densitometry and normalized to β-actin (n=3; mean ± SEM; ***p < 0.001, compared with cells without LiCl). The expressions of **(C)** RunX2 and **(D)** Osterix were examined by real-time PCR (The results are expressed as the mean ± SEM of three independent experiments. **p < 0.01, compared with cells without LiCl). **(E)** The expressions of RunX2 and Osterix were analysed by immunoblotting, β-actin was detected as a loading control. Levels of **(F)** RunX2 and **(G)** Osterix were quantified by densitometry and normalized to β-actin (Data are representative of three independent experiments and values are means ± SEM. ***p < 0.001, compared with cells without LiCl). **(H)** Calcium deposition was assessed by Alizarin red staining. **(I)** The Alizarin red staining was extracted and quantified, the wavelength was measured at 562 nm (Data are representative of three independent experiments and values are means ± SEM. ***p < 0.001, compared with cells without LiCl).

To further determine the contribution of Wnt/β-catenin signaling to NCAM-mediated osteoblast differentiation, the β-catenin level was increased by the Wnt pathway activator LiCl during osteogenic differentiation in Sh-NCAM MC3T3-E1 cells (**Figures 4A, B**). As shown in **Figures 4C, D**, inducing of β-catenin restored the mRNA expressions of *RunX2* and *Osterix* down-regulated by NCAM silencing. The protein levels of RunX2 and Osterix were also recovered in LiCl-treated Sh-NCAM MC3T3-E1 cells, compared with that in control cells (**Figures 4E–G**). Besides, Alizarin red staining and quantitative analysis revealed that β-catenin up-regulation reversed the lower accumulation of calcium content induced by NCAM silencing in Sh-NCAM MC3T3-E1 cells (**Figures 4H, I**). The results further confirmed the contribution of Wnt/β-catenin signaling to NCAM-mediated osteoblast differentiation.

### PI3K-Akt Signaling Contributes to NCAM-Mediated Osteoblast Differentiation

**Figure 5:**
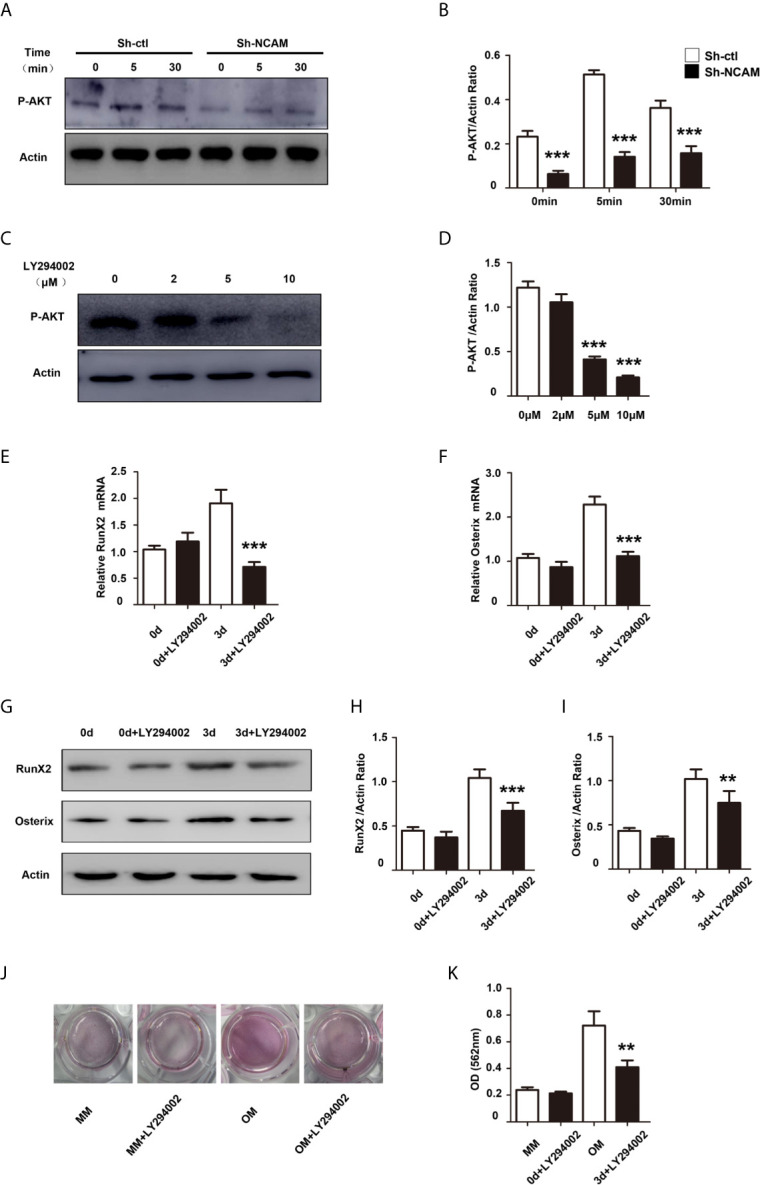
PI3K-Akt signaling contribute to NCAM-mediated osteoblast differentiation. **(A)** Sh-ctl and Sh-NCAM MC3T3-E1 cells were treated with differentiation media for 0, 5, and 30 min, the phosphorylation of Akt was analysed by immunoblotting. β-actin was detected as a loading control. **(B)** Level of phosphorylated Akt was quantified by densitometry and normalized to β-actin (n=3; mean ± SEM; ***p < 0.001, compared with control siRNA group). MC3T3-E1 cells were pretreated with the PI3K-Akt inhibitor LY294002. **(C)** The expression of phosphorylated Akt was analysed and **(D)** quantified by densitometry and normalized to β-actin (n=3; mean ± SEM; ***p < 0.001, compared with cells without LY294002).The expressions of **(E)** RunX2 and **(F)** Osterix were examined by real-time PCR (The results are expressed as the mean ± SEM of three independent experiments. ***p < 0.001, compared with cells without LY294002). **(G)** The expressions of RunX2 and Osterix were analysed by immunoblotting, β-actin was detected as a loading control. Levels of **(H)** RunX2 and **(I)** Osterix were quantified by densitometry and normalized to β-actin (Data are representative of three independent experiments and values are means ± SEM. **p < 0.01, ***p < 0.001, compared with cells without LY294002). **(J)** Calcium deposition was assessed by Alizarin red staining. **(K)** The Alizarin red staining was extracted and quantified, the wavelength was measured at 562 nm (Data are representative of three independent experiments and values are means ± SEM. **p < 0.01, compared with cells without LY294002).

The phosphatidylinositol 3’-kinase (PI3K)-Akt signaling plays a significant role in the osteoblast differentiation (18). To investigate the role of PI3K-Akt signaling in NCAM-mediated osteoblast differentiation, the phosphorylation of Akt was examined during osteogenic differentiation. The results showed that the Akt phosphorylation was significantly decreased in Sh-NCAM MC3T3-E1 cells as compared to Sh-ctl cells (**Figures 5A, B**). The Akt inhibitor LY294002 was applied to determine whether the NCAM-mediated Akt inhibition is involved in osteoblast differentiation. The data revealed that 10 μM of LY294002 was almost completely inhibited the phosphorylation of Akt (**Figures 5C, D**), and the levels of RunX2 and Osterix were significantly reduced in both mRNA expression (**Figures 5E, F**) and protein production (**Figures 5G–I**) during osteogenic differentiation in MC3T3-E1 cells. Alizarin red staining and quantitative analysis showed that the calcium deposition was also significantly decreased in LY294002-treated MC3T3-E1 cells (**Figures 5J, K**). These results indicated that Akt signaling is involved in NCAM-mediated osteoblast differentiation.

## Discussion

OP is a systemic skeletal disease characterized by low bone mineral density (19). Recent studies have shown that abnormality of osteoblast differentiation is one of the key factors leading to decreased bone mass (8, 20). In the present study, we revealed a novel role of NCAM in osteoblast differentiation. NCAM deficiency inhibits osteoblast differentiation in both mouse pre-osteoblast cells and mouse adult stem cells, in which Wnt/β-catenin and Akt signaling pathways play important roles.

MC3T3−E1, an osteoblast precursor cell line derived from mouse calvaria, has been widely used as a useful model for osteoblast differentiation research (21). Here, we explored the function of NCAM in osteoblast differentiation in MC3T3−E1 cells. During the process of osteoblast differentiation, *RunX2* and *Osterix* are key genes required for osteoblasts. Previous studies reported that Runx2-deficient (*Runx2^–/–^*) mice lack osteoblasts and bone formation (22, 23). Knockout of *Osterix* study in mice also demonstrated a complete lack of osteoblasts (24). Osterix and Runx2 initiate osteoblast differentiation by regulating the expression levels of various specific genes that characterize the osteogenic lineage, such as osteopontin, osteocalcin, bone sialoprotein, and alkaline phosphatase (25–28). In our study, we showed that NCAM silencing inhibited osteoblast differentiation as demonstrated by decreased induction of RunX2 and Osterix, and decreased calcium deposition in MC3T3−E1 cells. This novel role of NCAM was further corroborated in mouse MSCs, the differentiation of osteoblast was also inhibited in *Ncam^–/–^* MSCs. Our results demonstrated that either NCAM down-regulation in preosteoblast-like cells or NCAM deficiency in MSCs impaired osteoblast differentiation, indicating that NCAM plays a pivotal role in osteogenesis.

Wnt/β-catenin signaling, as the canonical Wnt pathway, is crucial for osteogenic lineage at early stages of osteogenic differentiation (29). Activation of Wnt/β-catenin signaling involves the binding of Wnt to surface receptors low-density lipoprotein receptor related protein 5/6 (LRP5/6) and Frizzled (30). In this study, we demonstrated that the β-catenin expression is decreased whereas the osteogenic markers and calcium deposition are recovered by Wnt activator in NCAM-silenced MC3T3−E1 cells. NCAM induces activation of FGFR and is an important interaction partner (31). Previous study (32) showed that NCAM-silencing represses β-catenin signaling *via* fibroblast growth factor receptor (FGFR) and glycogen synthase kinase-3β (GSK-3β). Other report has demonstrated that Wnt/β-catenin pathway reduces expression of NCAM through miR-30a-5p (33). These findings suggest that the effect of NCAM on Wnt signaling appears to bidirectional and indirect, which might be mediated by FGFR, GSK-3β or miR-30a-5p. PI3K/Akt signaling pathway was also found to play central role in osteoblast differentiation (34). We showed here that Akt activation is decreased in NCAM-silenced MC3T3−E1 cells and contributes to NCAM-mediated osteoblast differentiation. A previous study demonstrated that the critical osteogenic gene Runx2 is a target of β-catenin/TCF1 for the regulation of osteoblast differentiation and bone formation (35). Runx2 is also involved in Akt pathway activation, Runx2 and PI3K-Akt signaling are dependent on each other in the control of osteoblast differentiation, PI3K inhibitor inhibits DNA binding of Runx2 and Runx2-dependent transcription, and Runx2 also up-regulates expression of Akt and p85 and p110b subunits of PI3K (36). Another key osteogenic gene Osterix is a downstream osteoblastic gene of Runx2 (24, 37). These previous studies are consistent with our results indicating that NCAM silencing inhibits the expressions of osteogenic genes *Runx2* and Osterix by regulation of Wnt/β-catenin and PI3K-Akt signaling pathways.

OP and osteoarthritis (OA) are the two most common disorders of bone related to aging worldwide. OA is characterized by degeneration of cartilage and damage in subchondral and periarticular bone (38). Although some studies reported that OP may aggravates the degree of cartilage destruction in OA knee (39–41), the relationship between them is complex. In the present study, we found that NCAM silencing inhibits osteoblast differentiation, indicating that NCAM plays a key role in OP. We also previously demonstrated that NCAM deficiency increases the severity of cartilage damage in experimental OA (15). Our data provide NCAM as a novel target insight into the relationship between OA and OP, suggesting that overexpression of NACM can be used as a new potential strategy for the treatment of patients with OP and OA.

However, there are some limitations to the study, for example that it neither take into the role of NCAM in osteoblastogenesis *in vivo* studies, nor relates to further research on in-depth mechanism that how NCAM triggers the signaling pathways.

In conclusion, we demonstrate a novel role of NCAM in osteoblast differentiation in both pre-osteoblasts and MSCs. Wnt/β-catenin and PI3K-Akt signaling pathways contribute to NCAM-mediated osteoblast differentiation. The results support NCAM as a new regulator of osteoblast differentiation and a potential therapeutic target for OP treatment.

## Data Availability Statement

The original contributions presented in the study are included in the article/supplementary material. Further inquiries can be directed to the corresponding authors.

## Ethics Statement

The animal study was reviewed and approved by The Ethics Committee of Xinxiang Medical University.

## Author Contributions

H-JY and B-FC conceived and designed the experiments. B-FC, XF, Y-XG, LW, Y-FX, S-RL, and MW performed the experiments. XF, Y-XG, S-QJ and Z-WF analyzed the data. B-FC and H-JY wrote the manuscript. All authors contributed to the article and approved the submitted version.

## Funding

This work was supported by the grants from the National Natural Science Foundation of China (No. U1704186, 81771336 and 81671226), Key Scientific Research Project of Colleges and Universities in Henan Province (No. 19A310020), and Henan Neural Development Engineering Research Center for Children Foundation (SG201909).

## Conflict of Interest

The authors declare that the research was conducted in the absence of any commercial or financial relationships that could be construed as a potential conflict of interest.

## References

[1] van der Spoel E van Vliet NA van Heemst D. Viewpoint on the Role of Tissue Maintenance in Ageing: Focus on Biomarkers of Bone, Cartilage, Muscle, and Brain Tissue Maintenance. Ageing Res Rev (2019) 56:100964. 10.1016/j.arr.2019.100964 31561015

[2] Gennari L Rotatori S Bianciardi S Nuti R Merlotti D. Treatment Needs and Current Options for Postmenopausal Osteoporosis. Expert Opin Pharmacother (2016) 17(8):1141–52. 10.1080/14656566.2016.1176147 27055223

[3] Awasthi H Mani D Singh D Gupta A. The Underlying Pathophysiology and Therapeutic Approaches for Osteoporosis. Med Res Rev (2018) 38(6):2024–57. 10.1002/med.21504 29733451

[4] Wang Y Tao Y Hyman ME Li J Chen Y. Osteoporosis in China. Osteoporos Int (2009) 20(10):1651–62. 10.1007/s00198-009-0925-y 19415374

[5] Yang TL Shen H Liu A Dong SS Zhang L Deng FY. A Road Map for Understanding Molecular and Genetic Determinants of Osteoporosis. Nat Rev Endocrinol (2020) 16(2):91–103. 10.1038/s41574-019-0282-7 31792439 PMC6980376

[6] Takayanagi H. Osteoimmunology: Shared Mechanisms and Crosstalk Between the Immune and Bone Systems. Nat Rev Immunol (2007) 7(4):292–304. 10.1038/nri2062 17380158

[7] Xie Y Zhang L Xiong Q Gao Y Ge W Tang P. Bench-to-Bedside Strategies for Osteoporotic Fracture: From Osteoimmunology to Mechanosensation. Bone Res (2019) 7:25. 10.1038/s41413-019-0066-7 31646015 PMC6804735

[8] Qu H Li T Jin H Zhang S He B. Silent Mating Type Information Regulation 2 Homolog (SIRT1) Influences Osteogenic Proliferation and Differentiation of MC3T3-E1 Cells Via Regulation of Mir-132-3p. Med Sci Monit (2019) 25:2289–95. 10.12659/MSM.912392 PMC6451357 30923307

[9] Ronn LC Hartz BP Bock E. The Neural Cell Adhesion Molecule (NCAM) in Development and Plasticity of the Nervous System. Exp Gerontol (1998) 33(7-8):853–64. 10.1016/S0531-5565(98)00040-0 9951628

[10] Ronn LC Berezin V Bock E. The Neural Cell Adhesion Molecule in Synaptic Plasticity and Ageing. Int J Dev Neurosci (2000) 18(2-3):193–9. 10.1016/S0736-5748(99)00088-X 10715574

[11] Weinhold B Seidenfaden R Rockle I Muhlenhoff M Schertzinger F Conzelmann S. Genetic Ablation of Polysialic Acid Causes Severe Neurodevelopmental Defects Rescued by Deletion of the Neural Cell Adhesion Molecule. J Biol Chem (2005) 280(52):42971–7. 10.1074/jbc.M511097200 16267048

[12] Buhring HJ Treml S Cerabona F de Zwart P Kanz L Sobiesiak M. Phenotypic Characterization of Distinct Human Bone Marrow-Derived MSC Subsets. Ann N Y Acad Sci (2009) 1176:124–34. 10.1111/j.1749-6632.2009.04564.x 19796240

[13] Crigler L Robey RC Asawachaicharn A Gaupp D Phinney DG. Human Mesenchymal Stem Cell Subpopulations Express a Variety of Neuro-Regulatory Molecules and Promote Neuronal Cell Survival and Neuritogenesis. Exp Neurol (2006) 198(1):54–64. 10.1016/j.expneurol.2005.10.029 16336965

[14] Yang HJ Xia YY Wang L Liu R Goh KJ Ju PJ. A Novel Role for Neural Cell Adhesion Molecule in Modulating Insulin Signaling and Adipocyte Differentiation of Mouse Mesenchymal Stem Cells. J Cell Sci (2011) 124(Pt 15):2552–60. 10.1242/jcs.085340 21730021

[15] Cheng BF Lian JJ Yang HJ Wang L Yu HH Bi JJ. Neural Cell Adhesion Molecule Regulates Chondrocyte Hypertrophy in Chondrogenic Differentiation and Experimental Osteoarthritis. Stem Cells Transl Med (2020) 9(2):273–83. 10.1002/sctm.19-0190 PMC6988767 31742919

[16] Olofsson CS Hakansson J Salehi A Bengtsson M Galvanovskis J Partridge C. Impaired Insulin Exocytosis in Neural Cell Adhesion Molecule-/- Mice Due to Defective Reorganization of the Submembrane F-actin Network. Endocrinology (2009) 150(7):3067–75. 10.1210/en.2008-0475 PMC2703535 19213846

[17] Etheridge SL Spencer GJ Heath DJ Genever PG. Expression Profiling and Functional Analysis of Wnt Signaling Mechanisms in Mesenchymal Stem Cells. Stem Cells (2004) 22(5):849–60. 10.1634/stemcells.22-5-849 15342948

[18] Ghosh-Choudhury N Abboud SL Nishimura R Celeste A Mahimainathan L Choudhury GG. Requirement of BMP-2-induced Phosphatidylinositol 3-Kinase and Akt Serine/Threonine Kinase in Osteoblast Differentiation and Smad-dependent BMP-2 Gene Transcription. J Biol Chem (2002) 277(36):33361–8. 10.1074/jbc.M205053200 12084724

[19] Kanis JA. Diagnosis of Osteoporosis. Osteoporos Int (1997) 7 Suppl 3:S108–16. 10.1007/BF03194355 9536315

[20] Armas LA Recker RR. Pathophysiology of Osteoporosis: New Mechanistic Insights. Endocrinol Metab Clin North Am (2012) 41(3):475–86. 10.1016/j.ecl.2012.04.006 22877425

[21] Xu CP Sun HT Yang YJ Cui Z Wang J Yu B. ELP2 Negatively Regulates Osteoblastic Differentiation Impaired by Tumor Necrosis Factor Alpha in MC3T3-E1 Cells Through STAT3 Activation. J Cell Physiol (2019) 234(10):18075–85. 10.1002/jcp.28440 PMC6618314 30847950

[22] Komori T Yagi H Nomura S Yamaguchi A Sasaki K Deguchi K. Targeted Disruption of Cbfa1 Results in a Complete Lack of Bone Formation Owing to Maturational Arrest of Osteoblasts. Cell (1997) 89(5):755–64. 10.1016/s0092-8674(00)80258-5 9182763

[23] Otto F Thornell AP Crompton T Denzel A Gilmour KC Rosewell IR. Cbfa1, a Candidate Gene for Cleidocranial Dysplasia Syndrome, is Essential for Osteoblast Differentiation and Bone Development. Cell (1997) 89(5):765–71. 10.1016/s0092-8674(00)80259-7 9182764

[24] Nakashima K Zhou X Kunkel G Zhang Z Deng JM Behringer RR. The Novel Zinc Finger-Containing Transcription Factor Osterix is Required for Osteoblast Differentiation and Bone Formation. Cell (2002) 108(1):17–29. 10.1016/s0092-8674(01)00622-5 11792318

[25] Cheng A Genever PG. SOX9 Determines RUNX2 Transactivity by Directing Intracellular Degradation. J Bone Miner Res (2010) 25(12):2680–9. 10.1002/jbmr.174 20593410

[26] Fu H Doll B McNelis T Hollinger JO. Osteoblast Differentiation In Vitro and In Vivo Promoted by Osterix. J BioMed Mater Res A (2007) 83(3):770–8. 10.1002/jbm.a.31356 17559111

[27] Choi YH Han Y Jin SW Lee GH Kim GS Lee DY. Pseudoshikonin I Enhances Osteoblast Differentiation by Stimulating Runx2 and Osterix. J Cell Biochem (2018) 119(1):748–57. 10.1002/jcb.26238 28657691

[28] Komori T. Regulation of Proliferation, Differentiation and Functions of Osteoblasts by Runx2. Int J Mol Sci (2019) 20(7):1694. 10.3390/ijms20071694 PMC6480215 30987410

[29] Maupin KA Droscha CJ Williams BO. A Comprehensive Overview of Skeletal Phenotypes Associated With Alterations in Wnt/beta-catenin Signaling in Humans and Mice. Bone Res (2013) 1(1):27–71. 10.4248/BR201301004 26273492 PMC4472092

[30] Krishnan V Bryant HU Macdougald OA. Regulation of Bone Mass by Wnt Signaling. J Clin Invest (2006) 116(5):1202–9. 10.1172/JCI28551 PMC1451219 16670761

[31] Kiselyov VV Skladchikova G Hinsby AM Jensen PH Kulahin N Soroka V. Structural Basis for a Direct Interaction Between FGFR1 and NCAM and Evidence for a Regulatory Role of ATP. Structure (2003) 11(6):691–701. 10.1016/s0969-2126(03)00096-0 12791257

[32] Liu R Shi Y Yang HJ Wang L Zhang S Xia YY. Neural Cell Adhesion Molecule Potentiates the Growth of Murine Melanoma Via β-Catenin Signaling by Association With Fibroblast Growth Factor Receptor and Glycogen Synthase Kinase-3β. J Biol Chem (2011) 286(29):26127–37. 10.1074/jbc.M111.237297 PMC3138257 21628472

[33] Wang Z Dai X Chen Y Sun C Zhu Q Zhao H. MiR-30a-5p is Induced by Wnt/beta-catenin Pathway and Promotes Glioma Cell Invasion by Repressing NCAM. Biochem Biophys Res Commun (2015) 465(3):374–80. 10.1016/j.bbrc.2015.08.007 26255203

[34] Guntur AR Rosen CJ. The Skeleton: A Multi-Functional Complex Organ: New Insights Into Osteoblasts and Their Role in Bone Formation: The Central Role of PI3Kinase. J Endocrinol (2011) 211(2):123–30. 10.1530/JOE-11-0175 PMC3348869 21673026

[35] Gaur T Lengner CJ Hovhannisyan H Bhat RA Bodine PV Komm BS. Canonical WNT Signaling Promotes Osteogenesis by Directly Stimulating Runx2 Gene Expression. J Biol Chem (2005) 280(39):33132–40. 10.1074/jbc.M500608200 16043491

[36] Fujita T Azuma Y Fukuyama R Hattori Y Yoshida C Koida M. Runx2 Induces Osteoblast and Chondrocyte Differentiation and Enhances Their Migration by Coupling With PI3K-Akt Signaling. J Cell Biol (2004) 166(1):85–95. 10.1083/jcb.200401138 15226309 PMC2172136

[37] Nishio Y Dong Y Paris M O’Keefe RJ Schwarz EM Drissi H. Runx2-mediated Regulation of the Zinc Finger Osterix/Sp7 Gene. Gene (2006) 372:62–70. 10.1016/j.gene.2005.12.022 16574347

[38] Goldring SR Goldring MB. Changes in the Osteochondral Unit During Osteoarthritis: Structure, Function and Cartilage-Bone Crosstalk. Nat Rev Rheumatol (2016) 12(11):632–44. 10.1038/nrrheum.2016.148 27652499

[39] Wang CJ Huang CY Hsu SL Chen JH Cheng JH. Extracorporeal Shockwave Therapy in Osteoporotic Osteoarthritis of the Knee in Rats: An Experiment in Animals. Arthritis Res Ther (2014) 16(4):R139. 10.1186/ar4601 24994452 PMC4095692

[40] Calvo E Castaneda S Largo R Fernandez-Valle ME Rodriguez-Salvanes F Herrero-Beaumont G. Osteoporosis Increases the Severity of Cartilage Damage in an Experimental Model of Osteoarthritis in Rabbits. Osteoarthr Cartil (2007) 15(1):69–77. 10.1016/j.joca.2006.06.006 16861013

[41] Stewart A Black A Robins SP Reid DM. Bone Density and Bone Turnover in Patients With Osteoarthritis and Osteoporosis. J Rheumatol (1999) 26(3):622–6. 10090173

